# Effects of *Lactobacilli acidophilus* and/or spiramycin as an adjunct in toxoplasmosis infection challenged with diabetes

**DOI:** 10.1016/j.fawpar.2023.e00201

**Published:** 2023-06-27

**Authors:** Enas A. El Saftawy, Safaa A. Turkistani, Hadel M. Alghabban, Emad A. Albadawi, Basma EA Ibrahim, Suzan Morsy, Mohamed F. Farag, Nashwah S. Al Hariry, Rania Y. Shash, Aly Elkazaz, Noha M. Amin

**Affiliations:** a Medical Parasitology Department, Faculty of Medicine, Cairo University, Cairo, Egypt; b Medical Parasitology Department, Faculty of Medicine, Armed Forces College of Medicine, Cairo, Egypt; c Fakeeh College for Medical Sciences, Jeddah 21134, Saudi Arabia; d Department of Biochemistry and Molecular Medicine, College of Medicine, Taibah University, Saudi Arabia; e Department of Anatomy, College of Medicine, Taibah University, Saudi Arabia; f Physiological Sciences Department, Fakeeh College for Medical Sciences, Saudi Arabia; g Pathological Sciences Department, Fakeeh College for Medical Sciences, Saudi Arabia; h Medical Physiology Department, Armed Forces College of Medicine, Cairo, Egypt; i Faculty of Medicine, Department of Pathology, Suez University, Egypt; j Medical Microbiology and Immunology Department, Faculty of Medicine, Cairo University, Cairo, Egypt; k Pediatric Department, Faculty of Medicine, Cairo University, Cairo, Egypt; l Department of Clinical Pharmacology, Alexandria, Egypt; m Faculty of Medicine, Cairo University, Egypt

**Keywords:** Diabetes, Probiotics, Spiramycin, IL-17A, PD-1, Claudin-1

## Abstract

The current study assessed the anti-parasitic impact of probiotics on *Toxoplasma gondii* infection either solely or challenged with diabetes in Swiss albino mice. The study design encompassed group-A (diabetic), group-B (non-diabetic), and healthy controls (C). Each group was divided into infected-untreated (subgroup-1); infected and spiramycin-treated (subgroup-2); infected and probiotic*-*treated (subgroup-3); infected and spiramycin+ probiotic-treated (subgroup-4). Diabetic-untreated animals exhibited acute toxoplasmosis and higher cerebral parasite load. Overall, various treatments reduced intestinal pathology, improved body weight, and decreased mortalities; nevertheless, probiotic + spiramycin exhibited significant differences. On day 7 post-infection both PD-1 and IL-17A demonstrated higher scores in the intestine of diabetic-untreated mice compared with non-diabetics and healthy control; whereas, claudin-1 revealed worsening expression. Likewise, on day 104 post-infection cerebral PD-1 and IL-17A showed increased expressions in diabetic animals. Overall, treatment modalities revealed lower scores of PD-1 and IL-17A in non-diabetic subgroups compared with diabetics. Intestinal and cerebral expressions of IL-17A and PD-1 demonstrated positive correlations with cerebral parasite load. In conclusion, toxoplasmosis when challenged with diabetes showed massive pathological features and higher parasite load in the cerebral tissues. Probiotics are a promising adjunct to spiramycin by ameliorating IL-17A and PD-1 in the intestinal and cerebral tissues, improving the intestinal expression of claudin-1, and efficiently reducing the cerebral parasite load.

## 1 Introduction

*Toxoplasma gondii* is a worldwide endemic intracellular apicomplexan protozoan that can invade almost all nucleated cells and can infect numerous warm-blooded mammals (Lambert and Barragan, 2010). *T. gondii* appeared to be interrelated to diabetes mellitus (DM) as it had been considered an inducer of Type I DM by damaging the pancreatic β cells to prohibit insulin production (Shirbazou et al., 2013); besides, its role in Type 2 DM by inducing chronic low-grade inflammatory reactions (Nosaka et al., 2016; Li et al., 2018). Along the same line, DM especially in uncontrolled cases is associated with immune suppression (Ahmadikia et al., 2021) that increases the risk of *T. gondii* seroprevalence (Lewis et al., 2015).

However, studies that describe the accurate effects of toxoplasmosis and its associating criteria in DM are limited (Majidiani et al., 2016). Interestingly, the gastrointestinal manifestations in this risk group of patients are usually attributed to autonomic neuropathy common in diabetes (Bytzer et al., 2001) ignoring the role of infectious diseases. Indeed, prior studies underscored parasitic misdiagnosis in diabetic children (Rady et al., 2019) and adult patients (Poorkhosravani et al., 2019). Therefore, the figure of the inflammatory cascade in *T. gondii* infection challenged with DM appeared to be an interesting research point (Akinbo et al., 2013).

Claudin-1 is a tight junction protein that controls selective permeability and para-cellular diffusion inside an epithelium. Yet, Liu et al. (2009) deduced that enteric pathogens can affect the expression of claudin proteins. Therefore, intestinal permeability increases and the trans-epithelial resistance becomes reduced due to microbial infections (Poritz et al., 2011). IL-17A is a crucial cytokine that preserves the integrity of the enteric mucosa by moderating its interaction with the neighboring commensals and guarding against their invasion (Amatya et al., 2017). In addition, IL-17A induces the production of pro-inflammatory cytokines that affect the humoral and cellular immune responses against enteric pathogens (Chen and Kolls, 2017). PD-1 is a checkpoint protein and an inhibitory receptor related to the CD-28 family in NK cells, macrophages, T cells, B cells, and even the regulatory T cells (Gong et al., 2018). Makuku et al. (2021) demonstrated that changes in PD-1 expression affect both innate and adaptive immunity. It is worth mentioning that former studies related to IL-17A (Kumar et al., 2016; Douzandeh-Mobarrez and Kariminik, 2019) and PD-1 (Nakazawa et al., 2004; Scandiuzzi et al., 2014; Song et al., 2015), showed wide conflictions regarding the real role of these proteins during inflammation (Robertson et al., 2016; Beswick et al., 2018; Douzandeh-Mobarrez and Kariminik, 2019).

Another interesting point is the effective role of the available therapeutics for toxoplasmosis involving trimethoprim, sulfamethoxazole, sulfadiazine, clindamycin, and pyrimethamine, which are specific for the tachyzoite stage of the parasite. Yet, Periti et al. (1993) determined several adverse effects related to these antibiotics. This indicated the need for new or adjuvant management to improve immunity against parasite replication while protecting the integrity of the host's health (Salas-Lais et al., 2020).

We inquired if the application of probiotics in food products guards against inflammation due to acute toxoplasmosis in DM. In addition, the impact of this line of biotherapy on the enteric and cerebral inflammatory microenvironments is another need for research. The World Health Organization and Food and Agriculture Organization defined probiotics as bacteria that when applied in satisfactory quantities improve the fitness of the host (Reid, 2005) being related to the host metabolism (El Saftawy et al., 2021a, El Saftawy et al., 2021b). These bacteria can be viable (Reid, 2005) or dead (Taverniti and Guglielmetti, 2011). Probiotics act by stimulating the local enteric mucosa and systemic immunity; thus, the term ‘immuno-biotic’ (Clancy, 2003). Further analysis of the inflammatory biomarker is necessary to define the precise role of probiotics in the pathogenesis of this parasite.

In this study, we describe the influence of probiotics with or without spiramycin in toxoplasmosis either solely or challenged with diabetes in Swiss albino mice. We quantitatively evaluated the in situ production of IL-17A and PD-1 in the immune cells infiltrating the intestinal and cerebral tissues; the expression of claudin-1 protein in the intestinal tight junctions, and the parasite load in the cerebral tissues.

## 2 Methodology

### 2.1 Animals

A total of 90 Swiss albino male mice 10–12 weeks of age, 60–80 g were used. The animals were laboratory-bred, known to be pathogenic-free, and were obtained from the Animal House related to Theodor Bilharz Research Institute, Egypt.

### 2.2 Ethical statement

The experiment was recorded as CU/III/F/22/23 by the Institutional Animal Care and Use Committee, Cairo University. Animal care followed the “Guidelines for the Care and Use of Laboratory Animals” and involved well-adjusted diet formulation, all hygienic settings, a notice of dead animals, habituated temperature (32 °C) and humidity, and 12 h dark:12 h light cycles. Blood glucose and hemoglobin A1c (HbA1c) were evaluated (Du et al., 2013; Liu et al., 2019).

### 2.3 Experimental design and study groups

In this experiment, 90 mice were incorporated and divided into three groups: a diabetic group (A) (40 mice), a non-diabetic group (B)(40 mice), and a control group (C) (10 mice). Each of the diabetic (A) and non-diabetic (B) groups was subdivided into four subgroups (1,2,3, and 4). Each subgroup involved 10 mice as in Table 1:

**Table 1:** Distribution of the experimental groups.

Experimental groups	Condition
Group A. Infected diabetic mice	
Subgroup 1	Infected and non-treated
Subgroup 2	Infected and treated with spiramycin
Subgroup 3	Infected and treated with probiotics
Subgroup 4	Infected and treated with spiramycin + probiotics
Group B. Infected non-diabetic mice	
Subgroup 1	Infected and non-treated
Subgroup 2	Infected and treated with spiramycin
Subgroup 3	Infected and treated with probiotics
Subgroup 4	Infected and treated with spiramycin+probiotics
Group C. Negative control (healthy mice)	

### 2.4 Induction of diabetes

To induce diabetes, streptozotocin dissolved in citrate buffer was injected intraperitoneal in a dose of 55 mg/kg body weight for five successive days. Hyperglycemia was assessed at least 3 times throughout the 2nd week after initiating drug administration. Diabetes was considered positively induced when blood glucose was greater than 275 mg/dL in three consecutive measurements. A dose of 0.2 units of intermediate-acting insulin was administrated S.C. 3 times/week to avoid extreme weight loss in diabetic animals while preserving hyperglycemia and glycosuria (Furman, 2015).

### 2.5 Parasite inoculation

Strain ME-49 of *T. gondii* was attained from the National Research Center, Giza, Egypt. The cysts were obtained from infected mice slaughtered by cervical dislocation. Brains were extracted in sterile Falcon tubes and were homogenized in Hank's balanced salt solution. To induce intestinal pathology, the infective inoculum was adjusted into 10^2^ oocysts/2 ml i.e. each 20 μl with 1 oocyst in a wet mount. In the current experiment, the infection was initiated using an oesophageal tube by a suspension of 0.25 ml containing 15–20 cysts (El Saftawy et al., 2020). Mice were euthanized one-week post-infection (P.I.) for pathological assessment of the bowels (Johnson and Sayles, 1997) whereas the cerebral tissues were investigated on day 104 P.I. (El Saftawy et al., 2020).

### 2.6 Spiramycin treatment

The antibiotic was obtained from Sigma-Aldrich (USA) and dosing solutions were performed with distilled water and delivered to the mice perioral via a 22G feeding needle (Braintree Scientific, Inc., USA) after a12 hour fasting interval. The determined dose was 200 mg/kg/day 15 min P.I. for three consecutive days (Etewa et al., 2018).

### 2.7 Probiotic

To determine the effects of *Lactobacillus acidophilus* on immune enhancement mice were fed 10^9^ viable L. *acidophilus* /d for 14 d prior to the experiment. *L. acidophilus* capsules, Natrol *Acidophilus* Probiotic, composed of 10^9^ live cultures per capsule were purchased from pharma stocks in Cairo.

### 2.8 Histopathological examination

Serial sections of the ileum l region were assessed microscopically to determine inflammation in the lamina propria and alterations in the villous mucosa. Histopathological alterations were classified as 0 (no), 1 (mild), 2 (moderate), and 3 (severe) (Erben et al., 2014) where each subgroup demonstrated an average score for pathological changes.

### 2.9 Quantification of the parasite in the cerebral tissues

Using Olympus compound microscopy and low magnification (x4 objective) serial images were obtained for the H&E stained cerebral tissue-cut sections. Images of brain tissues of each mouse per subgroup were introduced in the Image-J software, where the grid tool was manipulated to enumerate *Toxoplasma* cysts within squares with definite pixel area. The mean number of parasites per mean pixel area per subgroup was calculated using the following formula (El Saftawy et al., 2020).

$\frac{C/N}{A/N}$C: no. of *T.gondii* cysts; N: no. of squares; A: pixel area

### 2.10 Processing of cerebral and intestinal tissues

Tissues collected from all animals were preserved in 10% formalin for 24 h; thereafter, tissues were fixed in paraffin wax for sectioning and mounting. Tissue sections of 5 μm were prepared for hematoxylin and eosin stain and immunohistochemistry staining (El Saftawy et al., 2020).

### 2.11 Immunohistochemistry (IHC) staining

Expressions of IL-17A, PD-1, and claudin-1 protein were assessed using primary monoclonal antibodies of rabbit anti-human origin: anti-IL-17A antibody # ab214588, anti-PD-1 antibody (PDCD1/1410R) # ab218475, and anti-claudin-1 antibody (EPRR18871) #ab211737 respectively. The main stocks of primary antibodies were diluted at 1: 200 with 20 mmol/l TBS, pH 7.4 (10 mmol/l CaCl_2_, 0.1% NaN_3_, and 1% BSA), and applied to the tissues. Thereafter, the tissue sections were incubated at room temperature with the biotinylated anti-polyvalent secondary antibody (sheep-origin) for ten minutes to bind with the formerly applied primary antibody. The final steps involved the addition of the di-amino-benzidine tetra-hydrochloride (DAB) solution (#AEX080) for 15 min., washing with distilled water, 70% ethanol for 1 min, and again with distilled water. The whole process was in accordance with the guidelines of the manufacturer. Negative controls were set in an identical protocol, excluding the consumption of the primary antibody (El Saftawy et al., 2022).

### 2.12 The immune-reactivity scoring of IHC

Expressions of IL-17A and PD-1 in the immune cells infiltrating intestinal and cerebral tissues in addition to the claudin protein in the intestinal tight junctions were all assessed. In the intestine, the evaluated biomarkers were considered positive if there was brownish immunostaining where the immune reactive cells were counted in 5 representative high-power fields HPFs (×400). The results were recorded as the average number of cells/HPF for each mouse and for each designated subgroup of mice. In the cerebral tissues, immune staining was automatically evaluated in ten fields in each tissue cut section at low (10×) and high magnifications (100×) for area percentage (area %) and optical density (O.D.) consequently using Real-Time Quantitative Morphocytometry Leica Qwin Analyzer (LEICA Imaging System Ltd., Cambridge, England). All records were saved for further statistical analysis (El-Aal et al., 2015; El Saftawy et al., 2021a).

### 2.13 Statistical analysis

Statistics were performed using the statistical package for the Social Sciences (SPSS) version 28 (IBM Corp., Armonk, NY, USA). Quantitative variables were précised in mean and standard deviation. Categorical variables were determined in the form of frequencies (number of cases) and relative frequencies (percentages). Comparisons between groups were performed using unpaired *t*-test or analysis of variance (ANOVA) with multiple comparisons post hoc test in normally distributed quantitative variables while non-parametric Kruskal-Wallis test and Mann-Whitney test were used for non-normally distributed quantitative variables (Chan, 2003a). For comparing categorical data, Chi-square (χ2) test was performed. When the expected frequency is <5 Exact test was involved (Chan, 2003b). The Spearman correlation coefficient was used for correlations between quantitative variables (Chan, 2003c). *P*-values <0.05 were statistically significant.

## 3 Results

### 3.1 Weight loss, mortality rate, and intestinal necrosis

**Fig. 1:**
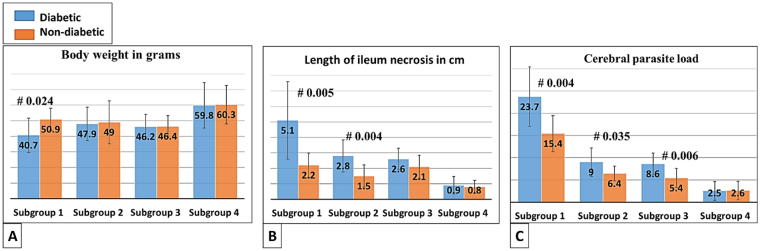
Bar charts showing a simple descriptive statistical analysis in mice either infected and challenged with DM (Group A) or solely infected (Group B). (A) Mean body weights on day 104 P.I., (B) mean length of ileum necrosis on day 7 P.I., and(C) mean cerebral parasite load on day 104 P.I. Note # refers to the p-value between diabetic and non-diabetic mice in each subgroup.

Body weights in the subgroups (A,1 & B,1) were reduced. However, it was improved in the subgroups (A4 & B4) on both day 7 P.I. and day 104 P.I.. (Fig. 1A). The total mortality rate on day 7 P.I. was more notable among the subgroups (A,1 & B,1), *p*-value <0.005. On day 104 P.I., the total mortality rate among treated subgroups was extremely reduced, p-value <0.05. On day 7 P.I., necrosis of the villi and mucosal cells significantly increased in subgroup A,1 and was reduced in probiotics sole therapy and combined treatment (Fig. 1, B). The healthy control showed long-term survival and intestinal necrosis was almost absent (*p*-value 0.02).

### 3.2 Histopathological alterations

Intestine

**Fig. 2:**
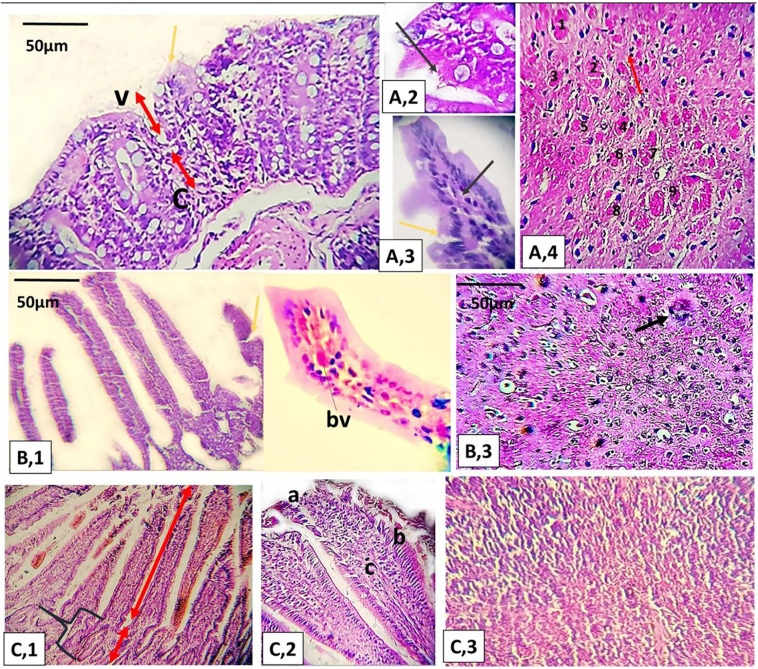
Hematoxylin and eosin stain of the tissue cut sections. Subgroups (A,1): (A,1) severe grade of ileitis with shortening and widening of villi (yellow arrow) and disrupted villus/crypt ratio (red arrows); (A,2) shortening of villi; (A,3) dense lymphoid aggregates and disrupted basal line (yellow arrow); and (A,4) cerebral tissue with variable-sized cysts (9 cysts) and chronic inflammatory cells (red arrows). Subgroup B,1: (B,1) moderate villous alterations, minor changes in the mucosal epithelium and basal nuclei (yellow arrow); (B,2) moderate clusters of inflammatory infiltrates in a villous and congestion (bv, blood vessel); and (B,3) *T.gondii* cyst in cerebral tissue (one cyst). Healthy control: (C,1) normal villous architecture and villus/crypt ratio, C: crypt depth; (C,2) component of villus showing a, normal brush border; b, basal nuclei; c, lamina propria; and (C,3) normal cerebral structure. (For interpretation of the references to colour in this figure legend, the reader is referred to the web version of this article.)

A pathological examination of the intestinal tissues revealed the increased intensity of ileitis in the subgroup (A,1) (Fig. 2). No changes were observed in the duodenum or jejunum. Subgroup (B,4) presented the weakest inflammatory response. Table 2 shows further details.

**Table 2:** Histological changes in the ileum of infected diabetic and nondiabetic subgroups.

Histological changes		Subgroup 1		Subgroup 2		Subgroup 3		Subgroup 4		P- value
		C*	%	C*	%	C*	%	C*	%	
Group A	Normal	0	0.0%	1	10.0%	0	0.0%	2	20.0%	< 0.001
	Mild	0	0.0%	1	10.0%	1	10.0%	7	70.0%	
	Moderate	7	70.0%	6	60.0%	3	60.0%	1	10.0%	
	Severe	3	30.0%	2	20.0%	6	40.0%	0	0.0%	
Group B	Normal	0	0.0%	2	20.0%	0	0.0%	2	20.0%	0.002
	Mild	4	40.0%	5	50.0%	2	20.0%	8	80.0%	
	Moderate	6	60.0%	3	30.0%	4	40.0%	0	0.0%	
	Severe	0	0.0%	0	0.0%	4	30.0%	0	0.0%	

P-value 0.714 0.132 0.02 1.

C* refers to counts.

Cerebral tissues

The parasite established niche-like cysts in the cerebral tissues (Fig. 2 A4 & B3). No remarkable histological alterations were detected except for inflammatory infiltrates that correlated in intensity with the surrounding parasite load.

Cerebral parasite load

Subgroups (A,1) had the highest mean parasite load per pixel area^2^ at day 104 P.I.. In the individualized therapies (either spiramycin or probiotics), the mean counts of parasite cysts declined (<*p*-value 0.05); yet, in combined treatment (spiramycin + probiotics), parasite cysts were almost scattered (Fig. 1, C).

### 3.3 Immune reactivity scores of IHC

Intestinal tissues

IL-17A

**Fig. 3:**
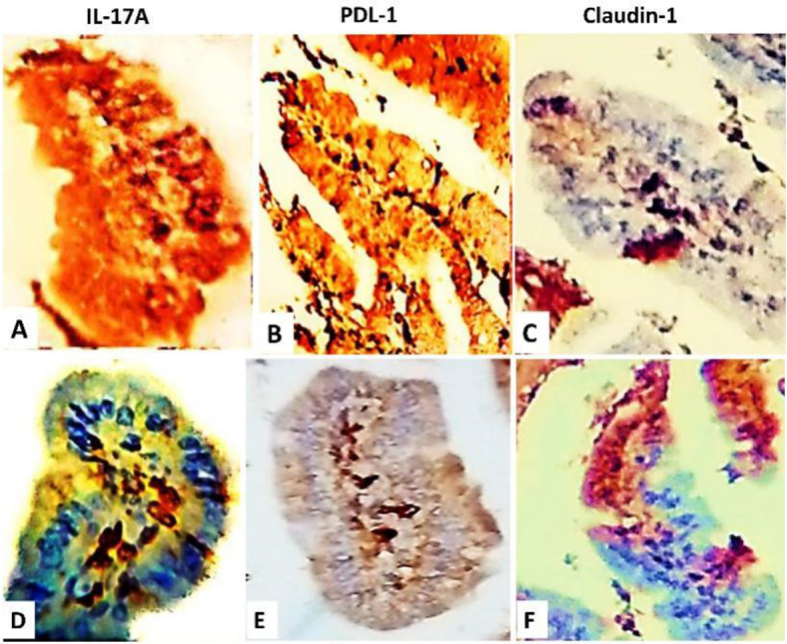
IHC staining of the ileum on day 7 P.I. in the respective subgroups A 1&B1. (A&D) show the cytoplasmic and nuclear expression of IL-17A. (B&E) show the membranous and cytoplasmic expression of PD-1. (C&F) show the membranous expression of claudin-1.

In subgroup (A,1), immune reactivity in the lymphoid aggregations significantly increased (Fig. 3A). In spiramycin, subgroup (A,2) showed declined cytoplasmic and nuclear immune reactivity. Exposure to probiotics immune reactive cells formed clusters in the subgroup (A3) and were scattered in subgroup B3, *p*-value <0.001. In combined therapy (probiotics + spiramycin), IL-17A immune reactive cells profoundly declined in both subgroups (A,4 & B,4) with a p-value of 0.006. In the healthy control, few IL-17A-immune positive cells were present. Details are shown in Table 3.

**Table 3:** Intestinal biomarkers in diabetic and non-diabetic subgroups.

Group	Intestinal biomarker	Group A		Group B		P-value
		Mean	SD	Mean	SD	
Subgroup 1	IL-17A	38.1	5.3	10.7	3.4	< 0.001
	PD-1	32.9	5.8	9.8	4.18	< 0.001
	Claudin-1	6.4	2.88	9.4	3.69	0.058
Subgroup 2	IL-17A	24.4	5.97	7	2.11	< 0.001
	PD-1	22.9	5.8	7	2.11	< 0.001
	Claudin-1	13.5	2.64	18.1	4.23	0.009
Subgroup 3	IL-17A	21.2	6.23	8.7	3.23	< 0.001
	PD-1	22.3	6.8	8.7	3.23	< 0.001
	Claudin-1	18.5	3.75	22.4	4.6	0.052
Subgroup 4	IL-17A	13.3	5.46	7.2	1.81	0.006
	PD-1	11.8	4.85	7.4	1.96	0.016
	Claudin-1	27.7	8	33.9	6.26	0.070

Interactions between intestinal biomarkers and cerebral parasite load.

PD-1

In untreated mice, PD-1 showed a predominant increase in the subgroup (A,1), with p-value <0.05; yet, in the subgroup (B, 1), the relative number of PD-1 positive cells showed a low value (Fig. 3B&E). Also, in single-treatment approaches, subgroups (A,2 & A,3) revealed an increased expression of PD-1. The subgroups (B,2 & B,3) showed a nearly constant or less number of PD-1-positive cells. In the combined therapy, PD-1 expression was dramatically reduced in the intestinal villi of the subgroup (A,4). In healthy controls, expression of PD-1 was scattered in the villi. Table 3 shows further details.

Claudin-1

In infected untreated subgroups (A,1 & B,1), claudin-1-positive epithelial cells were scattered and the relative numbers showed low mean values, *p*-value 0.058 (Fig. 3C &F). In spiramycin-treated subgroups, the relative number of immune-positive cells increased in the subgroup (B,2). In probiotics and combined therapy, the immune reactive epithelial cells were in clusters and increased in both diabetic and nondiabetic animals as shown in Table 3. In healthy control, claudin-1 was extensively expressed in the enteric epithelium.

**Fig. 4:**
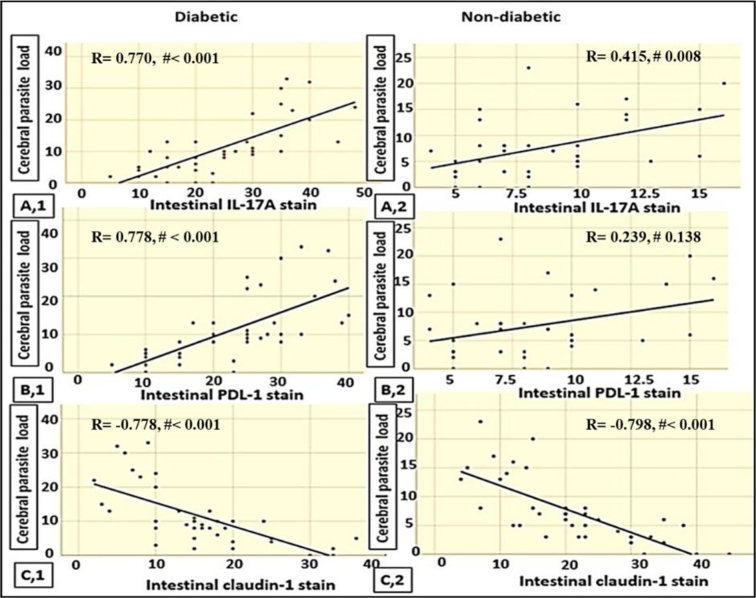
Correlation between the cerebral parasite load and intestinal biomarkers using the Spearman correlation coefficient. Open symbols and lines signify the total *T.gondii* infected populations either diabetic or nondiabetic. Note # refers to the *p*-value.

In diabetic mice, cerebral parasite load showed a strong positive correlation with IL-17A (Fig.4 A,1) and PD-1 (Fig.4 B,1). In nondiabetic mice, IL-17A was moderately correlated with the parasite load (Fig. 4 A,2). In both diabetic and nondiabetic mice, claudin-1 expression showed a significant inverse correlation with the parasite load (Fig.4 C,1&2).

Cerebral tissues

IL-17A

**Fig. 5:**
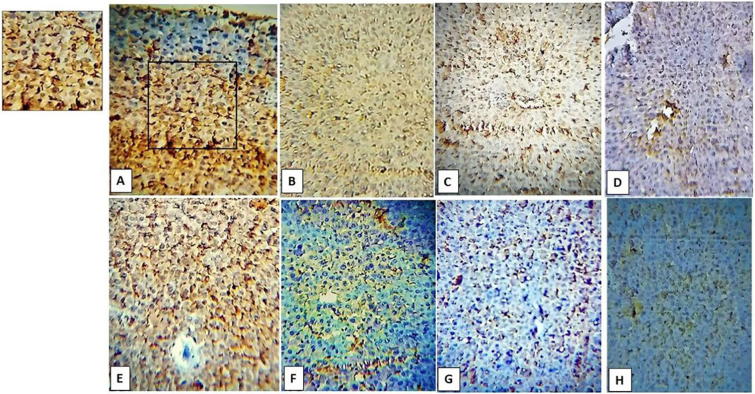
IHC staining of IL-17A in respective diabetic and nondiabetic cerebral tissues. A& E are infected untreated; B&F are spiramycin treated; C&G are probiotics treated and D & H are probiotic+spiramycin treated. Note the cropped figure on the upper left shows a nuclear and cytoplasmic expression of IL-17A.

In the case of infected untreated mice, IL-17A showed increased expression in the subgroup (A,1 & B,1). In spiramycin-treated subgroups, the IL-17A-positive immune cells were scattered in nondiabetic mice (subgroup B,2). In the case of probiotics and combined therapy, the relative number of IL-17A immune-positive cells showed low values in diabetic and non-diabetic animals (Fig. 5, Fig. 7). Healthy control revealed low or almost absent expression.

PD-1

**Fig. 6:**
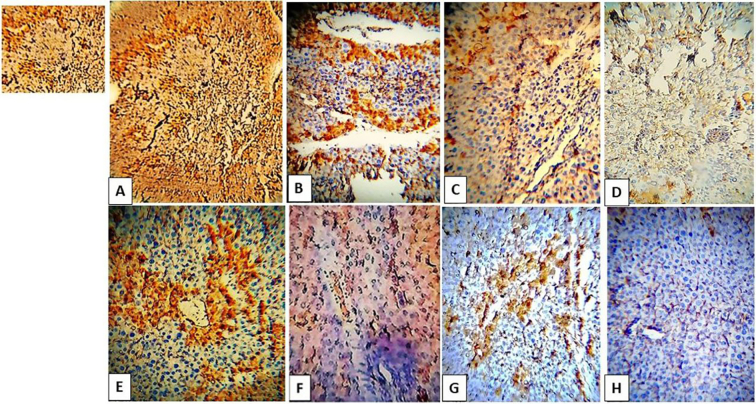
IHC staining of PD-1 in respective diabetic and non-diabetic cerebral tissues. A & E are infected untreated; B&F are spiramycin treated; C&G are probiotics treated and D&H are probiotic+spiramycin treated. Note the cropped figure on the upper left shows a *T.gondii* tissue cyst surrounded by PD-1 immune-reactive cells.

**Fig. 7:**
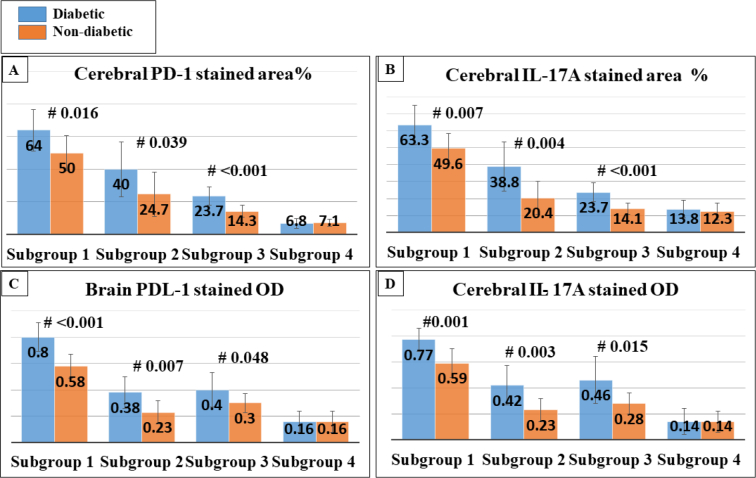
Representative quantification of IL-17A and PD-1 in diabetic and nondiabetic cerebral tissues.

PD-1 showed high immune positivity in the subgroup (A1&B1). In individualized therapies, PD-1 immune-positive cells were scattered both diabetic and nondiabetic mice. In combined therapy, the immune reactivity of PD-1 was reduced and almost absent (Fig. 6, Fig. 7). Healthy control showed low expression values.

Interactions between cerebral biomarkers and cerebral parasite load.

Collectively, statistics showed strong positive relationships between parasite load and the expressions of IL-17A and PD-1 on the cerebral tissues (*p*-value <0.001) (Table 4).

**Table 4:** Correlation coefficient analysis between Th17 and PD-1 expression and cerebral parasite burden.

	Biomarker		R-value
Group A	IL-17A	O.D.	0.736
		area %	0.764
	PD-1	O.D.	0.743
		area %	0.816
Group B	IL-17A	O.D.	0.669
		area %	0.731
	PD-1	O.D.	0.638
		area %	0.827

## 4 Discussion

We observed that toxoplasmosis triggered a significant loss of weight in untreated subgroups. Likewise, in prior studies, *Toxoplasma*-infected mice were found to lose muscle mass and fail to gain visceral adiposity (Dvorakova-Hortova et al., 2014; Hatter et al., 2018). The increased mortality among diabetic mice suffering comorbidity with acute toxoplasmosis (day 7 P.I.) reflects the aggrieving effect of the parasite. Recent studies suggested that *T. gondii* co-morbidity with other diseases, for example, COVID-19 (Roe, 2022) and HIV/AIDS (Mboera et al., 2019) was tremendously lethal. In this study, despite the significant effect of spiramycin in alleviating the death rate due to toxoplasmosis, probiotics were considerable to provide similar outcomes irrelevant to the presence of diabetes. Prior studies determined the beneficial effect of probiotics to alleviate complications of diabetes (Sun et al., 2020), glucose levels, histological alterations (Abdelazez et al., 2018), metabolism (Kocsis et al., 2020), and the disturbed intestinal microenvironment, for example, the outgrowth of *Clostridia* spp*.* (Hatter et al., 2018); henceforth improving cachexia.

Acute toxoplasmosis exhibited massive necrosis of the villi and mucosal cells in the ileum. Regarding a prior study, *T.gondii* triggers histologic alterations mediated by CD4+ T cells in a pattern similar to Crohn's disease (Liesenfeld, 2002). In the case of diabetes, Larsen et al. (2010) revealed that hyperglycemia alters microbiomes and induces inflammatory reactions that contribute to intestinal pathology. In the current study, histological changes improved significantly when treated with probiotics + spiramycin in both diabetic and nondiabetic mice. In regard to former studies, probiotics can modify host gene expression (Delzenne et al., 2013) and stimulate the production of anti-inflammatory cytokines such as IL-10 (Shen et al., 2018; Chang et al., 2017). Abdelazez et al. (2018) displayed the significant effect of probiotics on boosting histological healing in diabetes.

Cerebral tissues showed a higher parasite load in the infected untreated diabetics compared to infected untreated nondiabetic animals. This might be attributed to the debilitated cell-mediated immunity particularly T cells and phagocytic cells due to diabetes whereas *T.gondii* is an opportunistic intracellular protozoan (Hamamci and Acikgoz, 2019). In human studies, higher *T. gondii* seroprevalence was shown in diabetic patients compared to their healthy controls (Lewis et al., 2015; Li et al., 2018). In contrast to our results, Alvarado-Esquivel et al. (2017) deduced a lack of association between diabetes and toxoplasmosis. To our surprise, the combination treatment reduced parasite burden to the least values in both diabetic and non-diabetic mice. Konstantinovic et al. (2019) described those *Toxoplasma* therapies that can cross the blood-brain barrier and lessen the count of *T.gondii* cysts as “ideal treatment from the pharmacokinetic point of view”.

Intestinal expression of IL-17A increased following *T.gondii* infection. IL-17A in *T. gondii* infection plays a role in the recruitment of neutrophils to the sites of infections (Kelly et al., 2005). On the other side, prior studies deduced the reciprocal relationship between Th17 cells and the differentiation of Th1 cells and IFN-γ production; hence improving host survival (Moroda et al., 2017; Douzandeh-Mobarrez and Kariminik, 2019). Another study deduced that IL-17A is not involved in the intestinal inflammation triggered by *T. gondii* infection (Muñoz et al., 2009). Therefore, the protective functions of IL-17 against *T. gondii* remain indefinite (Washino et al., 2012).

Higher IL-17A expression was exhibited in the ileum of diabetic mice. Similar results were determined by Qiu et al. (2021). In the current model, there was an overall reduction in IL-17A expression following different therapeutic regimens. Grujić et al. (2005) deduced that spiramycin improves protection in acute toxoplasmosis; nevertheless, when we boosted immunity with probiotics in the combination treatment expression of IL-17A was reduced to the least values. Probiotics appeared to improve the microbial population and mucus secretion (Sagheddu et al., 2020) consecutively alleviating alterations induced by antibiotic treatment (McDonnell et al., 2021) and the parasite (Elsaftawy and Wassef, 2021). Also, probiotics reduce lipopolysaccharides that ligate TLR 2, and 4 to the antigen-presenting cells and enhance Th2/Th17 responses (Eslami et al., 2016); hence blunting unnecessary immune responses (Sagheddu et al., 2020).

PD-1 intestinal expression significantly increased in infected untreated mice. PD-1 interacts with CD80 to diminish the activation of T cells and cytokine production (Butte et al., 2007). Also, PD-1 interactions prevent CD8 T cell-mediated immune reactions. In this accordance, anti-PD-1 targeted therapy can lead to lethal enteritis, apoptosis of epithelial cells, and severe histological alterations (Presutti et al., 2007). On the contrary, a former study related the triggered pathway of PD-1 to inflammatory bowel disease (Robertson et al., 2016).

PD-1 intestinal expression significantly increased after infection in diabetic untreated mice compared with nondiabetic models. In prior studies, insulin was found to induce PD-1 expression (Heckl et al., 2021) that might attenuate the immune assault (Colli et al., 2018). In the current study, PD-1 expression showed a significant reduction in the combination treatment (spiramycin+probiotics) compared with untreated models. Regarding a prior study, this regulation of PD-L1 expression might occur in response to *Lactobacilli* and its interactions with mucosal professional antigen-presenting cells (Robertson et al., 2016) thus protecting against pathogens (Gopalakrishnan et al., 2018). In addition, Pons et al. (2021) deduced the immune modulatory effect of macrolides to sustain hemostasis. However, it was demonstrated that inhibition of the PD-1 pathway may disrupt intestinal tolerance, trigger CD8 T cells (Reynoso et al., 2009), disrupt Th2 polarization (Schwartz et al., 2017), and aggrieve diabetes (Yun et al., 2020; Kotwal et al., 2019). Interestingly, PD-1 activities maintain a healthy microbiome and regulate IgA production (Maruya et al., 2013). Thus, in the current study, the combined treatment appeared to be beneficial as it modulated PD-1 expression not profoundly but close to normal (healthy control).

The expression of claudin-1 in the ileum reduced significantly after infection with *T.gondii*. Beeman et al. (2012) determined that intestinal inflammation triggers the mislocalization of claudin and the disruption of tight junctions that subsequently trigger apoptosis in the epithelial cells. El Saftawy et al. (2020) and El Saftawy et al. (2021b) deduced that immunity against *T. gondii* infection can affect normal receptor expression and alters Bax/Bcl-2 ratio. The reduction in claudin-1 expression in the ileum was significantly profound in diabetic animals. Cani et al. (2007) speculated that in diabetes intestinal permeability increases thus facilitating the translocation of gut microbial products into the bloodstream causing endotoxemia.

Probiotics either sole therapy or combined with spiramycin showed a significant increase in the expression of claudin-1 with insignificant differences compared with the healthy uninfected control. Yoshida et al. (2018) and Chelakkot et al. (2018) demonstrated that probiotics increase gene expression of tight junction proteins and improve endotoxemia in diabetic models. Plovier et al. (2017) deduced that the outer membrane protein of these bacteria triggers the expression of the tight junction protein to recover the intestinal barrier (Carlsson et al., 2013).

The parasite load in the cerebral tissues correlated with the massive expression of Il-17A in the ileum after *T.gondii* infections in both diabetic and non-diabetic populations. Guiton et al. (2010) and Qiu et al. (2021) demonstrated the injurious effect of IL-17A on *T.gondii* infection and diabetes with no evident protection. Also, in the current work, diabetes was associated with increased PD-1 expression that positively correlated with cerebral parasite burden. This highlighted the immune evasive role of the PD-1 pathway during insulin treatment (Heckl et al., 2021). Concomitantly, claudin-1 showed a reduced expression that demonstrates increased permeability and systemic pathway of the parasite (Luettig et al., 2015).

Cerebral IL-17A expression was provoked during chronic toxoplasmosis wherein similar results were deduced by Guiton et al. (2010). IL-17A triggers the recruitment of neutrophils and the production of antimicrobial peptides against pathogens (Ishigame et al., 2009; Saijo et al., 2010). Yet, IL-17A has been related to neuro-inflammation (Stumhofer et al., 2006) as it causes disruption of tight junctions of the blood-brain barrier facilitating infiltration of Th17 cells to the cerebral tissues (Kebir et al., 2007). Yang et al. (2017) speculated that IL-17A upregulates the ATP-binding cassette subfamily-A member-1 in the endothelial cells of the blood-brain barrier. El Saftawy et al. (2020) and El Saftawy et al. (2021b) in former series of works related the provoked inflammatory cytokines to the possible development of dementia and Alzheimer's. Thus reduced expression of IL-17A in combined treatment (probiotics + spiramycin) appeared to be of protective impact. Guiton et al. (2010) suggested that neutralization of IL-17 has a partial protective effect against fatal inflammation induced by *T. gondii* infection.

Cerebral PD-1 expression increased immensely during chronic toxoplasmosis wherein similar results were deduced by Blackburn et al. (2009). In an experimental model of cerebral toxoplasmosis, upregulation of PD-1 characterized CD8 T cell exhaustion. Former studies deduced that CD8 T cells and IFN-γ are pivotal for resistance to chronic toxoplasmosis. Nevertheless, a series of works determined the exhaustion of CD8 T cells in chronic *T. gondii* infection (Bhadra et al., 2013; Bhadra et al., 2012), thus, these cells lose their potential capacity to proliferate and produce cytokines (Wherry, 2011). Interestingly, the blockade of the PD-1 pathway boosts CD8T cells and increases thrive of treated animals (Bhadra et al., 2011).

Irrespective of the presence of diabetes, the cerebral expression of IL-17A and PD-1 revealed a strong correlation with cerebral parasite load. In a prior study, IL-17 triggered the PD-1 pathway in cancer cells creating an immunosuppressive microenvironment (Wang et al., 2017) dominated by Treg cells whereas CD4 and Cd8 cells were inhibited (Ma et al., 2017). In parallel, our findings exhibit that promoted PD-1 and IL-17A expression contributed to increasing parasite burden.

## 5 Conclusion

Compared with healthy uninfected and infected untreated mice challenging toxoplasmosis with diabetes exhibited aggrieving intestinal necrosis and cerebral parasite load. There were a variety of expressions in IL-17A, PD-1, and claudin-1 between diabetic mice and non-diabetics. Collaboration of these agents appeared to break the intestinal barrier leading to a higher cerebral parasite load in toxoplasmosis challenged with diabetes. Of note, non-diabetics responded better to different therapeutic regimens with improved pathological features and local cellular responses. Probiotics+ spiramycin ultimately amended the expression of IL-17A and PD-1 in the intestine and brain tissues and claudin-1 in the small intestine when compared with single therapeutic regimens. In parallel, the cerebral load of the parasite was positively correlated with IL-17A and PD-1 expressions. Hence, we encourage combination therapy in the treatment of toxoplasmosis.

## Declaration of Competing Interest

The authors declare that there is no conflict of interest.
